# Adherence to Recommended Immunisation Schedules for Patients With Inflammatory Bowel Disease (IBD) on Biologics: A Retrospective Study at Our Lady's Hospital, Navan, Ireland

**DOI:** 10.7759/cureus.105024

**Published:** 2026-03-11

**Authors:** Adnan Khan, Maheen Shahab, Malik Maqsood Anwar

**Affiliations:** 1 Gastroenterology, Our Lady's Hospital, Navan, IRL; 2 Internal Medicine, Our Lady's Hospital, Navan, IRL

**Keywords:** biologic treatment, improving ibd patient vaccination, inflammatory bowel disease (ibd), vaccination policy, vaccine preventable diseases

## Abstract

Introduction

Patients with inflammatory bowel disease (IBD), particularly those receiving biologic therapies, are at increased risk of infections due to immunosuppression. Vaccinations play a vital role in reducing this risk, yet adherence to immunisation schedules remains suboptimal. This audit aimed to assess vaccination coverage among IBD patients on biologic therapies at Our Lady's Hospital, Navan, Ireland, and to identify areas for improvement.

Aim and objectives

The primary aim was to evaluate adherence to recommended immunisation schedules for IBD patients on biologics. Specific objectives were to assess uptake of vaccines including tetanus, diphtheria, and pertussis (Tdap), meningococcal, measles, mumps, and rubella (MMR), severe acute respiratory syndrome coronavirus 2 (SARS-CoV-2), influenza, herpes zoster, human papillomavirus (HPV), hepatitis B virus (HBV), hepatitis A virus (HAV), and varicella-zoster virus (VZV), to identify gaps in coverage, and to propose interventions to improve adherence.

Methods

A retrospective review of 24 patients with IBD receiving regular biologic infusions at the hospital's day ward was conducted. Data collected included demographics, type of IBD, biologic therapy details, and vaccination status. The assessment was based on the European Crohn's and Colitis Organisation (ECCO)-aligned immunisation recommendations. Immunity to VZV was accepted based on documented infection history or serological confirmation.

Results

Among the 24 patients reviewed, 14 had ulcerative colitis and 10 had Crohn's disease. Sixteen were male and eight female. Twenty patients were on infliximab and four on vedolizumab. Vaccination coverage was generally low: Tdap (5/11, 45.5%), meningococcal (1/4, 25%), MMR (1/2, 50%), SARS-CoV-2 (0/4, 0%), influenza (1/10, 10%), HPV (1/3 eligible females, 33.3%), HBV (2/15, 13.3%), and HAV (0/4, 0%). All six patients with VZV data showed immunity. No documentation was available for herpes zoster vaccination.

Conclusion

The audit revealed significant gaps in adherence to vaccination guidelines among IBD patients receiving biologics. These findings highlight the need for systematic interventions to improve vaccine uptake, such as routine immunisation status reviews during clinic visits, improved documentation practices, patient education on vaccine safety and necessity, and stronger collaboration between gastroenterologists and primary care providers. Educational materials should be provided during clinic or infusion appointments to address vaccine hesitancy and misinformation. Implementing these changes could reduce the burden of vaccine-preventable diseases in this vulnerable population.

## Introduction

Inflammatory bowel disease (IBD) patients, particularly those on biologic therapies, face increased susceptibility to infections due to immunosuppression. Large observational studies have demonstrated that immunosuppressive and biologic therapies are associated with an increased risk of opportunistic and vaccine-preventable infections in patients with IBD. Vaccinations are a cornerstone of infection prevention in this population. However, adherence to recommended immunisation schedules remains suboptimal. Guidelines from the European Crohn's and Colitis Organisation (ECCO) emphasise the importance of reviewing and updating immunisation status, ideally prior to initiating immunosuppressive therapy [1]. Data regarding vaccination adherence among biologic-treated IBD patients in Ireland remain limited, highlighting the need for local evaluation of preventive care practices within routine clinical settings.

## Materials and methods

Study design and setting

This was a retrospective clinical audit of vaccination practices conducted at the day ward of Our Lady's Hospital, Navan, Ireland. The audit covered a 12-month period from January 2024 to December 2024. The objective was to assess vaccination coverage among patients with IBD receiving biologic therapy, benchmarked against recommendations from the ECCO.

Study population

All adult patients (≥18 years) with a confirmed diagnosis of either Crohn's disease or ulcerative colitis who received regular biologic infusions during the study period were eligible for inclusion. A total of 24 eligible patients were identified during the study period and included in the audit.

Diagnosis of Crohn's disease or ulcerative colitis was based on documented clinical diagnoses within patient records supported by standard diagnostic investigations. The study population consisted of patients receiving intravenous biologic infusions through the hospital day ward infusion service, and all eligible patients during the study period were included.

Data collection

Patients were identified with the assistance of the hospital's IBD clinical nurse specialist and a senior house officer. A list of eligible patients was generated from the day ward infusion records. Vaccination data were then obtained from electronic laboratory systems and patient medical charts, and all information was entered into a structured Microsoft Excel database (Microsoft Corp., Redmond, WA, USA). For each patient, the following variables were extracted: demographic details including age and sex, type of IBD (Crohn's disease or ulcerative colitis), biologic agent and duration of therapy, and vaccination status for tetanus, diphtheria, and pertussis (Tdap), meningococcal, measles, mumps, and rubella (MMR), severe acute respiratory syndrome coronavirus 2 (SARS-CoV-2), influenza, herpes zoster, human papillomavirus (HPV), hepatitis B virus (HBV), hepatitis A virus (HAV), and varicella-zoster virus (VZV). Immunity to VZV was determined either through documented prior infection or by serological confirmation.

Audit standards

Audit standards were based on the 2021 ECCO guidelines on the prevention, diagnosis, and management of infections in IBD [1], which recommend systematic review and updating of immunisation status, particularly before initiating immunosuppressive or biologic therapy.

Outcome measures

The primary outcome was the proportion of patients appropriately vaccinated against each recommended pathogen. Secondary outcomes included the identification of gaps in vaccination coverage and the assessment of documentation quality.

Appropriate vaccination status was determined according to ECCO-aligned recommendations and available clinical documentation. This included receipt of recommended vaccines, documentation of completed vaccination series where applicable, and confirmation of immunity for VZV through either prior infection history or serological evidence.

Data analysis

Descriptive statistics were applied. Results were expressed as frequencies and percentages and are presented in both tabular and graphical formats.

Ethical considerations

This project was conducted as a clinical audit/service evaluation of existing practice assessing adherence to vaccination guidelines in routine clinical care. Approval to conduct the audit was obtained from the supervising consultant gastroenterologist. In accordance with local institutional policy, audits involving retrospective review of anonymised patient records do not require formal research ethics committee approval.

## Results

Patient demographics

**Figure 1:**
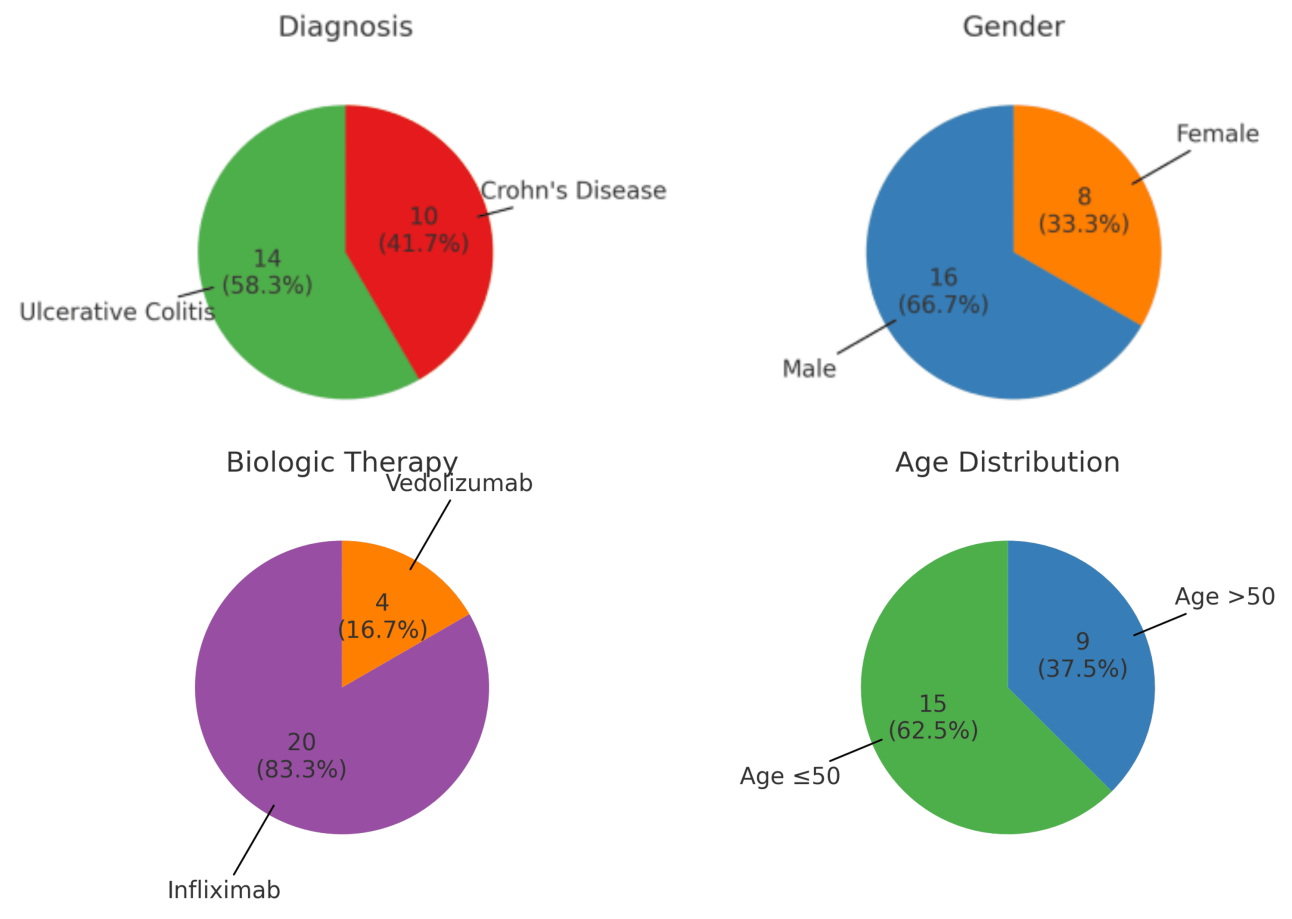
Patient demographics (n = 24). Distribution of patients by diagnosis, gender, biologic therapy, and age group. Frequencies (n) and percentages are shown within each pie slice.

Of the 24 patients included, 14 (58.3%) had ulcerative colitis and 10 (41.7%) had Crohn's disease. Sixteen patients (66.7%) were male, and eight (33.3%) were female. Twenty patients (83.3%) were receiving infliximab, and four (16.7%) were receiving vedolizumab. Nine patients (37.5%) were aged over 50 years. The demographic distribution of the cohort is shown in Figure 1.

Vaccination coverage

Overall vaccine uptake was low across the cohort. Only five of 11 patients with available records for Tdap had received the vaccine (45.5%). For the meningococcal vaccine, one of four patients (25%) had been vaccinated, while one of two patients with records for MMR vaccination was vaccinated (50%). None of the four patients with available documentation for SARS-CoV-2 vaccination had received it. For the annual influenza vaccine, only one of 10 patients (10%) was vaccinated. No records were available for herpes zoster vaccination. Among the three eligible female patients, one (33.3%) had received the HPV vaccine. For HBV, two of 15 patients (13.3%) were vaccinated, while none of the four patients with HAV records were vaccinated. All six patients with available VZV data demonstrated immunity. These findings are summarised in Table 1.

**Table 1:** Vaccination coverage among patients with IBD receiving biologic therapy (n = 24). The table summarises the number of eligible patients, those vaccinated, and the percentage coverage for each recommended vaccine. Tdap: tetanus, diphtheria, and pertussis; MMR: measles, mumps, and rubella; SARS-CoV-2: severe acute respiratory syndrome coronavirus 2; HPV: human papillomavirus; HBV: hepatitis B virus; HAV: hepatitis A virus; VZV: varicella-zoster virus; IBD: inflammatory bowel disease

Vaccine	Records available	Patients vaccinated	Compliance rate (%)
Tdap	11	5	45%
Meningococcal	4	1	25%
MMR	2	1	50%
SARS-CoV-2	4	0	0%
Annual influenza	10	1	10%
Herpes zoster	0	0	0%
HPV	3 (females only)	1	33.3%
HBV	15	2	13.3%
HAV	4	0	0%
VZV	6	All immune	100%

## Discussion

The audit conducted at Our Lady's Hospital, Navan, demonstrates significant deficiencies in adherence to recommended immunisation schedules among patients with IBD receiving biologic therapy. Only five of 11 patients with available Tdap vaccine records were vaccinated, along with one of four for meningococcal, one of two for MMR, and none of the four for SARS-CoV-2. Uptake of influenza (1/10), HPV (1/3), HBV (2/15), and HAV (0/4) vaccines was similarly low, despite the heightened infection risk associated with immunosuppressive treatment. These findings underscore a persistent gap in preventive care for this high-risk population.

The ECCO recommends that all patients with IBD receive appropriate vaccinations, ideally before the initiation of immunosuppressive therapy, and that their immunisation status be regularly reviewed and updated [1]. Tools such as the ECCO checklist have been developed to assist physicians during the initial evaluation. This checklist encompasses medical history, physical examination, laboratory assessments, and vaccination status. It also emphasises patient education and referrals to appropriate specialists, including those for gynecological screening and travel health. Vaccinations should ultimately be tailored to individual patient needs [2].

Similarly, the American College of Gastroenterology (ACG) and the Canadian Association of Gastroenterology (CAG) emphasise that all non-live vaccines are safe to administer during immunosuppression and should not be delayed. Live vaccines, in contrast, should be avoided once immunosuppressive or biologic therapy has commenced. Importantly, emerging evidence confirms that SARS-CoV-2 vaccination is both safe and immunogenic in IBD populations and does not increase the risk of disease flare [3-7].

Barriers to optimal immunisation in IBD include limited provider awareness, uncertainty regarding responsibility between primary and specialist care, patient misconceptions about vaccine safety, and fragmented access to immunisation records. All major guidelines advocate for gastroenterologists to play an active role in reviewing immunisation status, issuing clear recommendations to primary care, and employing tools such as vaccination checklists and electronic reminders. Provider recommendation remains one of the strongest predictors of vaccine uptake [3-7]. A comparative study further demonstrated that adherence to vaccination guidelines was significantly higher among IBD subspecialists compared with general gastroenterologists, underscoring the value of subspecialty expertise and IBD-focused practice in improving preventive care delivery [8].

The findings in this audit are consistent with other international studies. Similar deficiencies in vaccine uptake have been documented in Asian IBD cohorts, where insufficient coverage for hepatitis B, influenza, and pneumococcal vaccines has been observed [9,10]. Physician behaviour also contributes to this gap: in Italy, Macaluso et al. reported that although over 80% of gastroenterologists rated vaccination as "very important," only about half routinely recommended immunisation at diagnosis, with substantial variation across vaccine types [11]. In a large Spanish cohort, compliance with immunisation guidelines among IBD patients did not exceed 65% for any vaccine, and significant age-related differences were seen for MMR, VZV, influenza, and hepatitis A/B [12]. Similarly, a study of patients receiving anti-TNF therapy (predominantly infliximab) found that only 43 of 310 eligible individuals had received the recommended vaccinations, underscoring the gap between awareness and implementation. Although most physicians acknowledged the importance of pre-therapy immunisation, factors such as inadequate guideline dissemination, clinical workload, vaccine availability, and advanced disease state limited uptake [13].

Systematic interventions have been shown to substantially improve vaccination uptake in IBD patients. Patel and colleagues reported that barrier-oriented strategies, such as clinical reminders, vaccination prompts during infusions, or in-clinic vaccination programs, yielded the greatest impact on pneumococcal vaccine coverage (OR: 12.7; 95% CI: 2.2-72.6) compared to usual care [14]. Similarly, a recent study from China highlighted the critical role of combating misinformation, demonstrating that unreliable online information and fear of adverse effects were major barriers to vaccination among IBD patients, with a significant negative association between misinformation exposure and vaccine willingness [15].

In summary, this audit highlights a critical and addressable gap in preventive care for IBD patients on biologic therapy. This trend mirrors international studies, which have consistently reported suboptimal vaccination coverage among IBD patients, particularly those treated with immunosuppressive or biologic therapies. A coordinated, multidisciplinary approach is essential, incorporating proactive immunisation reviews at key clinical encounters, patient and provider education, integration of vaccination tools into care pathways, and improved communication between gastroenterology and primary care.

Despite these insights, the present audit is limited by its small sample size, reflecting a single-centre cohort that may not be generalisable to broader populations. Incomplete documentation within medical records may also have led to the underestimation of true vaccination coverage. Furthermore, the retrospective design restricted the evaluation of reasons for non-vaccination, such as patient refusal, contraindications, or logistical barriers. Nevertheless, these findings provide valuable insight into real-world gaps in preventive care for IBD patients on biologics and form a basis for targeted quality improvement initiatives.

To address these deficiencies, several strategies should be implemented. Regular review of immunisation status should be incorporated into routine IBD care, particularly prior to the initiation of biologic therapy. Patient education must be strengthened through the development and distribution of targeted materials during clinic and infusion visits, highlighting the safety and necessity of vaccines in this vulnerable population. Close collaboration between gastroenterologists and primary care providers is essential to ensure shared responsibility for vaccination delivery and follow-up.

Finally, a re-audit is planned within six months to evaluate the impact of these interventions. Key outcomes will include improvements in documentation, vaccine uptake, and patient education practices. This continuous quality improvement cycle will inform further strategies to optimise vaccination coverage and reduce the burden of preventable infections in patients with IBD on biologic therapies.

## Conclusions

This audit highlights marked deficiencies in adherence to recommended immunisation schedules among patients with IBD receiving biologic therapy. Despite clear international guidelines, vaccination coverage for key preventable infections remains suboptimal. These findings reinforce the need for systematic review of vaccination status as part of routine IBD management, along with patient education and improved collaboration between specialist and primary care services. Addressing these gaps has the potential to reduce preventable infections and improve long-term outcomes in this high-risk population.
